# Differentiating Damp‐Heat and Cold‐Damp Diarrhea in Rat Models via Gut Microbiota Dysbiosis and Short‐Chain Fatty Acid Profiling

**DOI:** 10.1002/mbo3.70164

**Published:** 2026-01-21

**Authors:** Hao Zhang, Xia Song, Wenwen Mi, Peng Ji, Yanming Wei, Yongli Hua

**Affiliations:** ^1^ Institute of Traditional Chinese Veterinary Medicine, College of Veterinary Medicine Gansu Agricultural University Lanzhou China

**Keywords:** cold‐damp diarrhea, damp‐heat diarrhea, gut microbiota, SCFAs

## Abstract

On the basis of gut microbiota and short‐chain fatty acids (SCFAs), this study aims to identify diagnostic biomarkers for damp‐heat diarrhea and cold‐damp diarrhea. Rat models of damp‐heat diarrhea and cold‐damp diarrhea were established. Changes in body weight, body temperature, food intake, water consumption, and the diarrhea index were recorded. ELISA was employed to detect levels of IL‐6, IL‐1β, TNF‐α, and IL‐10. Histological evaluations were conducted using H&E staining and AB‐PAS staining techniques. Transmission electron microscopy was utilized to observe ultrastructural changes in the colonic epithelium, while Western blot analysis was performed to assess the expression of Occludin, Claudin1, Claudin5, GPR41, GPR43, GPR109A, and NLRP3 in colon tissues. GC–MS analysis was carried out to determine the content of SCFAs in the cecal contents of rats; additionally, 16S rRNA sequencing was performed to analyze the composition of gut microbiota in these animals. Differential analysis methods were applied to evaluate similarities and differences in SCFAs profiles and gut microbiota between damp‐heat diarrhea and cold‐damp conditions. The body weight and food intake of rats with induced damp‐heat diarrhea or cold‐damp diarrhea significantly decreased over time as their diarrheal symptoms progressively worsened. However, following treatment with appropriate prescriptions tailored for each condition resulted in an improvement in diarrheal symptoms among the affected rats. In accordance with the “prescription‐based syndrome differentiation” theory, the rat experimental animal models of damp‐heat diarrhea and cold‐dampness diarrhea were successfully established. The models exhibited characteristic diarrheal symptoms alongside increased levels of inflammatory factors indicative of severe histopathological damage; there was also a notable reduction in tight junction protein expression observed across all models studied. Furthermore, the Firmicutes/Bacteroidota ratio showed a significant decrease. Interestingly, differences between damp‐heat diarrhea and cold‐damp diarrhea manifested as follows: Both modeling groups showed an increase in the relative abundance of *Lachnoclostridium* and *Marvinbryantia*. In the damp‐heat diarrhea group, the levels of *Lachnoclostridium* and *Marvinbryantia* were relatively low; however, these levels gradually increased after successful treatment. In contrast, in the cold‐damp diarrhea group, the trends of *Lachnoclostridium* and *Marvinbryantia* were opposite. Mucosal color has the potential for clinical diagnosis of damp‐heat diarrhea and cold‐damp diarrhea. Moreover, *Lachnoclostridium* and *Marvinbryantia* are potential biomarkers for distinguishing between damp‐heat diarrhea and cold‐damp diarrhea. However, the diagnostic basis and accuracy of *Lachnoclostridium* and *Marvinbryantia* biomarkers still need to be further validated.

## Graphical abstract

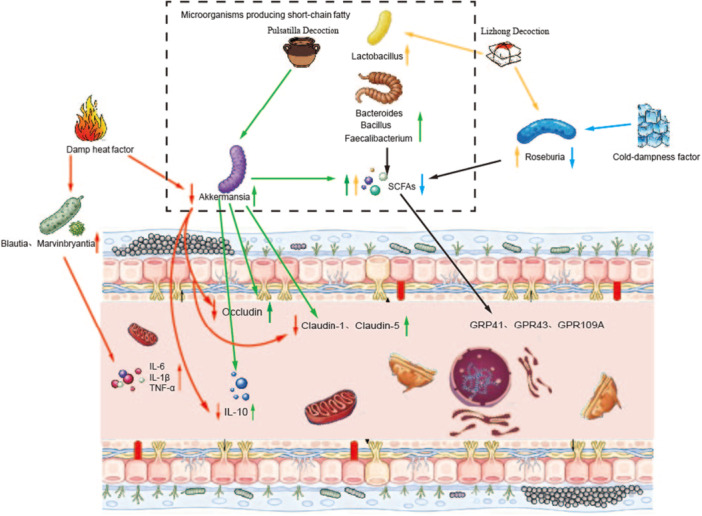

## 1 Introduction

Diarrhea is a symptom of a debilitating and potentially life‐threatening condition that affects the rectum and colon to varying extents, often associated with multiple underlying diseases. The global incidence of diarrhea is estimated to range from 3% to 20%, and this figure continues to rise. Factors such as inflammatory responses, damage to the intestinal epithelial barrier, dysregulation of gut microbiota, and immune responses all contribute to its pathophysiology (Chu et al. 2020). Epidemiological studies have indicated a rapid increase in the costs associated with treating diarrhea, significantly impacting patients' quality of life. Consequently, this condition poses a substantial challenge to global health efforts (Shankar and Durairaj 2024).

Current treatment strategies encompass symptom relief, prevention of related complications, dietary improvement, and promotion of mucosal healing. Pharmacological interventions include 5‐aminosalicylic acid, corticosteroids, immunomodulators, probiotics, and the promising therapy of fecal microbiota transplantation (Le Berre et al. 2023). Nevertheless, some patients may experience intolerance to drug side effects, such as allergic reactions and gastrointestinal symptoms (Ulcerative Colitis 2020). Despite the expansion of treatment options, 10%–20% of patients still necessitate proctocolectomy for the management of medically intractable diseases. Currently, total proctocolectomy can resolve colonic inflammation; however, postoperative complications are common, particularly pouchitis and fecal incontinence (Papasotiriou et al. 2023). Consequently, an increasing number of patients urgently require new therapeutic approaches, especially traditional Chinese medicine (TCM) therapies that have been practiced in Asia for thousands of years (Zheng et al. 2022; Hao et al. 2024). According to the principles of syndrome differentiation and treatment in TCM, diarrhea is classified into categories, such as damp‐heat diarrhea and cold‐damp diarrhea (Tang et al. 2021).

Damp‐heat diarrhea is often caused by staying in a damp and hot environment for a long time, with external damp‐heat pathogenic factors invading the human body from the surface. Long‐term consumption of spicy, greasy and fried foods leads to internal heat pathogenic factors in the intestines. Cold‐dampness diarrhea is caused by the combination of cold and dampness invading the human body, or by consuming raw and cold food, leading to the internal generation of cold. Damp‐heat diarrhea and cold‐damp diarrhea are prevalent syndromes encountered in clinical practice, both characterized by symptoms of diarrhea. The clinical presentation is complex and variable, which poses challenges in differentiating between these conditions and selecting appropriate treatment regimens. TCM utilizes distinct formulas tailored to address distinct syndrome. Consequently, there is an urgent need for biomarkers that can effectively delineate the differences between them. Reports suggest a close relationship between diarrhea and intestinal microbiota (L. Wang, Shao, et al. 2023; Z. Wang, Yang, et al. 2023); therefore, this study aims to identify biomarkers for the clinical diagnosis of damp‐heat diarrhea and cold‐damp diarrhea based on analyses of intestinal microbiota and short‐chain fatty acids (SCFAs). Pulsatilla decoction and Lizhong decoction (Lee et al. 2016) are established prescriptions used for the treatment of damp‐heat diarrhea and cold‐damp diarrhea, respectively (Hua et al. 2019, 2020). On the basis of the “Consensus opinion on TCM diagnosis and treatment of diarrhea,” the evaluation indicators of the rat models of damp‐heat diarrhea and cold‐dampness diarrhea were constructed (Wu et al. 2024). Pulsatilla decoction and Lizhong decoction were selected to verify the successful establishment of damp‐heat and cold‐damp diarrhea models. This study primarily aims to identify biomarkers for damp‐heat diarrhea and cold‐damp diarrhea derived from the gut microbiota.

## 2 Materials and Methods

### 2.1 Experimental Animals

SPF‐grade male Sprague‐Dawley (SD) rats, weighing 200 ± 5 g, were acquired from the Experimental Animal Center of Lanzhou Veterinary Research Institute (license approval number: SCXK(Gan)2020–0002). The care and use of animals adhered to the “Guidelines for the Management and Use of Experimental Animals” established by the Ministry of Science and Technology of China (2006), and were approved by the Gansu Agricultural University Animal Ethics Committee (GSAU‐Eth‐VMC‐2023–040). The animals were housed under controlled conditions at a temperature of 25°C, with humidity ranging from 40% to 50%, and subjected to a 12‐h light/dark cycle for an acclimatization period of 7 days.

### 2.2 Instruments and Reagents

ABI GeneAmp 9700 polymerase chain reaction (PCR) System; QuantiFluor‐ST Blue Fluorescence Quantitation System (Promega Corporation); Gas Chromatography (GC) (Model Agilent‐8890 GC, equipped with a Flame Ionization Detector), Chromatography Column (DB‐FFAP 30 mm × 0.25 mm, 0.25 μm), products of Agilent Technologies, USA; AL104 Electronic Balance, product of Shanghai Mettler‐Toledo Instruments Co. Ltd.; HITACHI‐CT15RE Vortex Mixer, product of Shanghai Kanghua Biochemical Instruments Manufacturing Factory; Shenzhen Lanjieke Technology Co. Ltd.; RM2235 Microtome, product of Leica, Germany; Olympus DP‐71, product of Olympus Corporation, Japan.

TransGen AP221‐02: TransStart FastPfu DNA Polymerase; AxyPrep DNA Gel Recovery Kit (AXYGEN Corporation); Tris–HCl Buffer Elution; TruSeq DNA Sample Prep Kit. SCFAs standards: acetic acid (GC purity ≥ 99.8%), propionic acid (GC purity ≥ 99.5%), isobutyric acid (GC purity ≥ 99.5%), butyric acid (GC purity ≥ 99.5%), isovaleric acid (GC purity ≥ 99.0%), valeric acid (GC purity ≥ 99.5%) all are high‐performance liquid chromatography grade (all standards purchased from Shanghai Macklin Biochemical Co. Ltd. (Shanghai, China). *N*‐butanol is of analytical reagent grade, purchased from Shandong Shuangshuang Chemical Co. Ltd. (Shandong, China). Ultrapure water is Wahaha purified water, cationic detergent sodium dodecyl sulfate (S8010), tetramethylethylenediamine (T8090), separating gel buffer (S1051), concentrated gel buffer (S1052), Tween 20 (T8220), Protein Marker Ⅳ (8–200 kDa) (G2083), purchased from Wuhan Servicebio Technology Co. Ltd. (Wuhan, China). TJP1 Rabbit pAb (bs‐1329R), purchased from Beijing Biosen Biotech Co. Ltd. (Beijing, China). Beta Actin (20536‐1‐AP), Occludin (13409‐1‐AP), E‐cadherin (20874‐1‐AP), horseradish peroxidase‐labeled goat antirabbit immunoglobulin G H + L (SA00001‐3), purchased from Proteintech Group Co. Ltd. (Wuhan, China). Nitrocellulose transfer membrane (BS‐PVDF‐22), interleukin 6 (IL‐6) (ml102828), IL‐10 (ml002813), tumor necrosis factor‐α (TNF‐α) (ml002859), IL‐1β (ml037361), enzyme‐linked immunosorbent assay (ELISA) kits purchased from Shanghai Enzyme‐linked Biotechnology Co. Ltd. (Shanghai, China). Ultramicro ATPase assay kit (Na^+^–K^+^ and Ca^2+^–Mg^2+^–ATPase) (A070‐6‐2) biochemical reagent kit purchased from Nanjing Jianchen Bioengineering Institute (Nanjing, China).

### 2.3 Preparation of Medicinal Materials

The herbs (Table 1) required for the study were purchased from the wholesale market of Yellow River herbs in Lanzhou City and identified by Professor Wei Yanming (the Department of Chinese Veterinary Medcine, College of Animal Medcine, Gansu Agricultural University). The herbs were crushed and soaked in distilled water for 1 h followed by decocting and filtration three times. The filtration was concentrated into 1 g/mL decoction at 60°C and preserved at 4°C.

**Table 1:** Composition of Chinese herbal formulas.

Senna aqueous	Dose (g)	Pulsatilla decoction	Dose (g)	Lizhong decoction	Dose (g)
*Cassia angustifolia* Vahl. (Fan Xie Ye)	30	*Pulsatilla chinensis* (Bge.) Regel (Bai Tou Weng)	30	*Zingiber officinale* Rosc. (Gan Jiang)	30
		*Phellodendron chinense* Schneid. (Huang Bai)	24	*Atractylodes macrocephala* Koidz. (Bai Zhu)	30
		*Fraxinus rhynchophylla* Hance. (Qin Pi)	24	*Codonopsis pilosula* (Franch.) Nannf. (Dang Shen)	30
		*Coptis chinensis* Franch. (Huang Lian)	12	*Glycyrrhiza uralensis* Fisch. (Zhi Gan Cao)	30

### 2.4 Animal Grouping

A total of 84 male SD rats (*n* = 84) were randomly divided into seven groups (*n* = 12/group): Control Normal Group (CON), Damp‐Heat Diarrhea Group (DHS), Cold‐Damp Diarrhea Group (CDS), Lizhong decoction Treatment for Damp‐Heat Diarrhea Group (LDH), Lizhong decoction Treatment for Cold‐Damp Diarrhea Group (LCD), Pulsatilla decoction Treatment for Damp‐Heat Diarrhea Group (PDH), and Pulsatilla decoction Treatment for Cold‐Damp Diarrhea Group (PCD).

### 2.5 Construction of Damp‐Heat Diarrhea and Cold‐Damp Diarrhea Models

**Figure 1:**
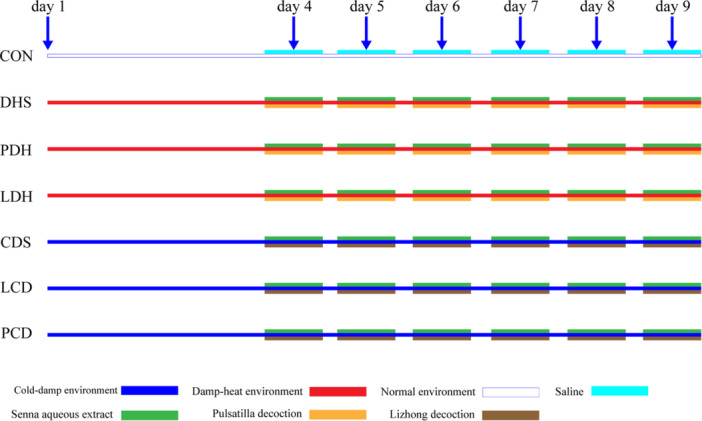
Schematic diagram of modeling. CDS, Cold‐Damp Syndrome; CON, control; DHS, Damp‐Heat Syndrome; LCD, Lizhong decoction Treatment for Cold‐Damp Diarrhea Group; LDH, Lizhong decoction Treatment for Damp‐Heat Diarrhea Group; PCD, Pulsatilla decoction Treatment for Cold‐Damp Diarrhea Group; PDH, Pulsatilla decoction Treatment for Damp‐Heat Diarrhea Group.

During the modeling process, rats had unrestricted access to water and food. The control (CON) group was maintained in an environment with a temperature of 25°C ± 2°C and humidity of 35% ± 5%. The CDS, PCD, and LCD groups were placed in an environment with a temperature of 4°C ± 2°C and humidity of 90% ± 5% for 8 h daily; rats in the DHS, PDH, and LDH groups were placed in an environment with a temperature of 34°C ± 2°C and humidity of 90% ± 5% for 8 h daily (Figure 1).

For the first 3 days, all groups except the CON group were acclimated in a temperature‐controlled device. On the evening of day four, the CON group received saline via gavage, while other groups were gavaged with Senna leaves (10 g/kg/d). The next morning, the CON, DHS, and CDS groups received saline again; LDH and LCD groups were given Lizhong decoction (4.375 g/kg/d), and PDH and PCD groups received Pulsatilla decoction (3.76 g/kg/d) for six consecutive days. On the afternoon of day 10, rats were anesthetized and exsanguinated through the abdominal aorta to prepare serum samples. The colon was fixed in 10% neutral formalin, while remaining intestinal and serum samples were stored at −80°C. Drug dosages are based on prestudy results.

On the 1st, 3rd, 5th, and 7th days of modeling, fecal samples from each group of rats were collected to assess fecal water content. Throughout the modeling period, body temperature, body weight, food intake, and water consumption were recorded daily at 8:00 AM. Rats in the CDS, LCD, and PCD groups had unrestricted access to ice water, whereas the remaining groups were provided with sterile water for free consumption.

### 2.6 General Behavior Observation and Diarrhea Severity Assessment in Rats

The behavioral characteristics of the rats were systematically observed and recorded at fixed daily intervals, alongside measurements of body temperature, body weight, food intake, and water consumption. The Disease Activity Index (DAI) was calculated as a composite score derived from all these parameters. To standardize assessments, the rats were induced to defecate at consistent times each day; the severity of diarrhea was graded accordingly. This included calculations for the rate of loose stools, average grade of loose stools, fecal water content, and diarrhea index.

Formulas are as follows:

(1) Rate of loose stools = Number of loose stools/Total number of stools × 100%, (2) Diarrhea index = Rate of loose stools × Average grade of loose stools, (3) Average grade of loose stools = Sum of all grades of loose stools/Number of loose stool occurrences, (4) Fecal water content, on the 1st, 3rd, 5th, 7th, and 9th days, feces were collected and weighed (M1), dried at 120°C for 12 h and weighed again (M2). Fecal water content (%) = [(Weight before drying (M1) − Weight after drying (M2))/Weight before drying (M1)] × 100%.

The grade of loose stools was divided into four levels based on the diameter of the filter paper soiled by the feces (Table 2) (Wu et al. 2024). Table 2 describes the classification criteria for fecal moisture content; no statistical analysis was performed on these criteria.

**Table 2:** Classification criteria for loose stool rate.

Water trail diameter (cm)	Stool rating
< 1	Level 1
1–1.9	Level 2
2–3	Level 3
> 3	Level 4

### 2.7 Histological Assessment and Alcian Blue‐Periodic Acid‐Schiff (AB‐PAS) Staining

Colons from all groups of rats were removed, washed with saline, fixed in 4% paraformaldehyde, washed with water, dehydrated with ethanol, and embedded in paraffin. Paraffin sections of rat colon tissue were conventionally deparaffinized in water and stained with AB‐PAS staining kit (Wuhan Saiwei Bio, China) according to the instructions. Standard histological pathology scoring.

### 2.8 Transmission Electron Microscopy (TEM) Observation of Rat Colon Tissue Ultrastructure

Colon tissue was fixed in 3% glutaraldehyde. Samples were washed three times with phosphate‐buffered saline (pH 7.2–7.4). Samples were exposed to 1% osmium tetroxide for 1.5 h, then dehydrated. Samples were infiltrated with Epon 812 resin and stained with a double staining method (uranyl acetate and lead citrate). A JEM‐1400 PLUS (JEOL, Japan) transmission electron microscope was used for TEM.

### 2.9 Cytokine Detection

Blood is collected, and serum is prepared. Commercial ELISA kits were used to measure the levels of IL‐6, IL‐1β, TNF‐α, and IL‐10 in serum according to the manufacturer's instructions.

### 2.10 Collection of Cecal Contents, Extraction of Bacterial DNA, Illumina MiSeq Sequencing, and Data Processing

Rats were swiftly euthanized using cervical dislocation. From each group, six rats were selected, and cecal contents were collected on a sterile bench. The samples were subsequently stored at −80°C before sequencing.

Microbial DNA was extracted (HiPure Stool DNA Kits, Magen, Guangzhou, China). The V3‐V4 hypervariable regions of the bacterial 16S ribosomal RNA (rRNA) gene were amplified using an ABI GeneAmp 9700 PCR thermal cycler (ABI, CA, USA) with primer pairs 338F (5′‐ACTCCTACGGGAGGCAGCAG‐3′) and 806R (5′‐GGACTACHVGGGTW TTAAT‐3′). PCR products were extracted from a 2% agarose gel and purified using the AxyPrep DNA Gel Extraction Kit (Axygen Biosciences, CA, USA), and quantified using the Quantus Fluorometer (Promega, USA). The purified PCR products were prepared for sequencing using the NEXTFLEX Rapid DNA‐Seq Kit: (1) adapter ligation, (2) removal of adapter dimers using magnetic beads, (3) enrichment of library templates using PCR amplification, and (4) recovery of PCR products using magnetic beads to obtain the final library. Sequencing was performed on the Illumina PE300/PE250 platform (Shanghai Majorbio Bio‐Pharm Technology Co. Ltd.).

Then, UPARSE 7.1 clustered the optimized sequences into operational taxonomic units (OTUs) at a 97% sequence similarity level (Edgar 2013). Each OTU representative sequence was taxonomically analyzed against the 16S rRNA gene database Silva v138 using RDP Classifier 2.2. Bacterial classification and data analysis were performed on the Majorbio Cloud platform (www.majorbio.com) online platform.

### 2.11 Western Blot Analysis

Rat colon tissue was homogenized with RIPA lysis buffer at low temperature and lysed as per the manufacturer's instructions. The mixture was centrifuged at 4°C to obtain total protein samples, which were quantified using a BCA assay kit. Proteins were separated by 10% sodium dodecyl sulfate–polyacrylamide gel electrophoresis, transferred onto a 0.45‐µm polyvinylidene fluoride membrane, blocked with 7% skim milk at room temperature, and incubated overnight with primary antibody at 4°C. Afterward, horseradish peroxidase‐labeled secondary antibody was added for 1 h at room temperature, followed by detection with enhanced chemiluminescent solution. Immunoblot bands were quantified using ImageJ software.

### 2.12 Gas Chromatography–Mass Spectrometry (GC–MS) Detection of SCFAs

The injection volume was 1 μL, and the samples were injected with a 10:1 split ratio. Acetic acid, propionic acid, butyric acid, isobutyric acid, valeric acid, isovaleric acid, and caproic acid standards were weighed and prepared into eight concentration gradients of 0.1, 0.5, 1, 5, 10, 20, 50, and 100 μg/mL with ethyl acetate. A final concentration of 500 μM–600 μL standard solution with 4‐methylvaleric acid as the internal standard was added, mixed, and subjected to GC–MS detection with an injection volume of 1 μL and a split ratio of 10:1.

Samples were frozen on ice, and 40 mg of the sample was placed in a 2‐mL glass centrifuge tube. Add 1200 μL of 0.5% phosphoric acid, resuspend, vortex, and mix for 2 min; centrifuge at 14,000*g* for 10 min, take 800 μL of the supernatant, add an equal volume of ethyl acetate for extraction, vortex for 2 min, centrifuge at 14,000*g* for 15 min; take 600 μL of the upper organic phase, add 500 μM 4‐methylvaleric acid as the internal standard, mix, add to the sample vial, inject 1 μL, with a split ratio of 10:1, and inject in portions. MSD ChemStation software was used to plot peak area and retention time. Standard curves were plotted to calculate the content of SCFAs in the samples.

### 2.13 Statistical Analysis

Experimental data were analyzed using IBM SPSS Statistics 24.0 (IBM Corporation, Armonk, NY, USA). Continuous data that met the assumptions of normality and homogeneity were compared using one‐way analysis of variance. For data that did not meet these assumptions, nonparametric tests such as the Kruskal–Wallis test were used. Statistical significance was set at *p* < 0.05.

## 3 Results

### 3.1 General Symptom Observation

**Figure 2:**
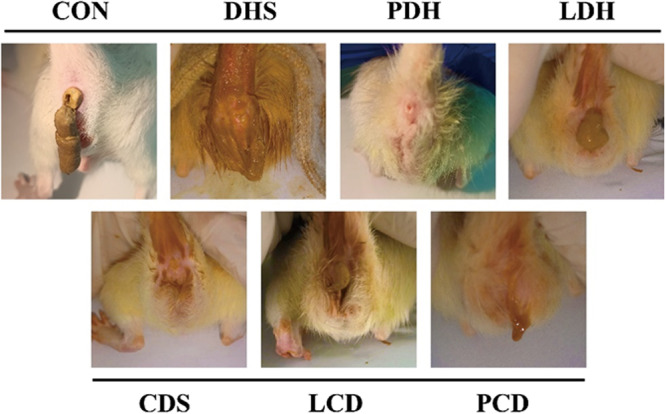
The contamination around the anus of rats. CDS, Cold‐Damp Syndrome; CON, control; DHS, Damp‐Heat Syndrome; LCD, Lizhong decoction Treatment for Cold‐Damp Diarrhea Group; LDH, Lizhong decoction Treatment for Damp‐Heat Diarrhea Group; PCD, Pulsatilla decoction Treatment for Cold‐Damp Diarrhea Group; PDH, Pulsatilla decoction Treatment for Damp‐Heat Diarrhea Group.

**Figure 3:**
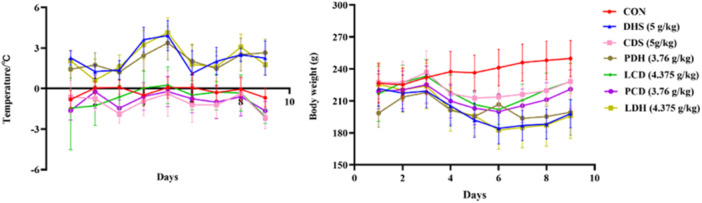
Trend charts of body temperature differences and body weight changes in each group of rats. *Note:* The temperature difference = posttreatment body temperature − pretreatment body temperature. CDS, Cold‐Damp Syndrome; CON, control; DHS, Damp‐Heat Syndrome; LCD, Lizhong decoction Treatment for Cold‐Damp Diarrhea Group; LDH, Lizhong decoction Treatment for Damp‐Heat Diarrhea Group; PCD, Pulsatilla decoction Treatment for Cold‐Damp Diarrhea Group; PDH, Pulsatilla decoction Treatment for Damp‐Heat Diarrhea Group.

Clinical symptoms are illustrated in Figure 2. After the perianal area of the rats in the CON group was cleaned and the feces were formed and molded, the anal temperature of the rats in the damp‐heat diarrhea (DHS) group increased, and the perianal area was contaminated by mucopurulent feces, leading to anal loosening in Figure 3. The rats in the PDH group were in good spirits, had smooth defecation and clean perianal area, while the rats in the LDH group had difficulty defecating, with constipation and watery stools alternating. In the cold‐dampness diarrhea (CDS) group, rats were still clustered and drowsy, with contracted abdominal walls, decreased anal temperature, watery stools, knotted perianal hair, and slight contamination. The mental state of the rats in the LCD group recovered, their activity levels increased, the anal temperature approached normal, and they alternated between mucus stool and constipation during defecation. Rats in the PCD group were clustered, with low rectal temperature, insufficient activity, contracted abdominal walls and mucus stools.

**Figure 4:**
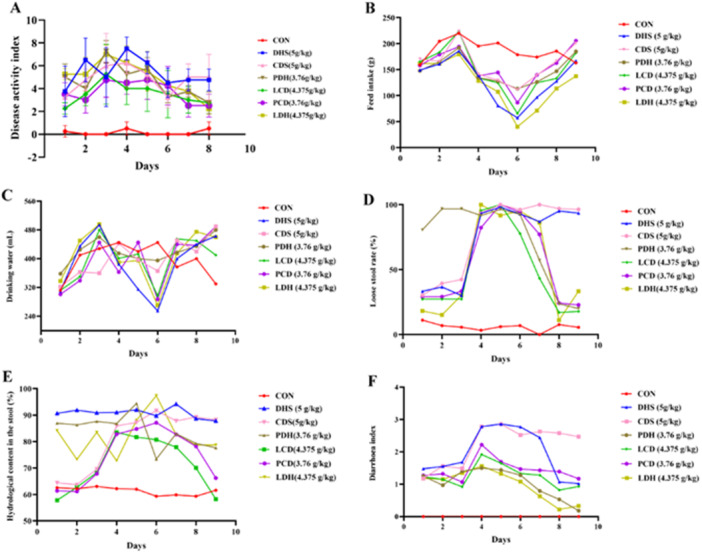
Trends in basic data changes of rats. (A) DAI score, (B) trends in food intake changes, (C) trends in water intake changes, (D) diarrhea rate, (E) fecal water content, and (F) diarrhea index. CDS, Cold‐Damp Syndrome; CON, control; DAI, Disease Activity Index; DHS, Damp‐Heat Syndrome; LCD, Lizhong decoction Treatment for Cold‐Damp Diarrhea Group; LDH, Lizhong decoction Treatment for Damp‐Heat Diarrhea Group; PCD, Pulsatilla decoction Treatment for Cold‐Damp Diarrhea Group; PDH, Pulsatilla decoction Treatment for Damp‐Heat Diarrhea Group.

Overall, the food and water intake of the CON group remained stable, whereas other groups exhibited a trend of initially decreasing followed by an increase (Figure 4A–C). The DAI index for the CON group consistently stayed at a low level, in contrast to the other groups whose DAI indices demonstrated an initial increase followed by a gradual decline. In terms of fecal water content, diarrhea rate, and diarrhea index, rats in the CON group maintained low levels throughout. Conversely, both the diarrhea rate and fecal water content in rats from the DHS and CDS groups increased significantly and sustained high levels. Additionally, rats in the PDH, LDH, LCD, and PCD groups displayed trends characterized by an initial rise followed by a decrease in their diarrhea rates, fecal water content, and diarrhea indices (Figure 4D–F).

### 3.2 Histopathological Evaluation and Ultrastructural Observation

**Figure 5:**
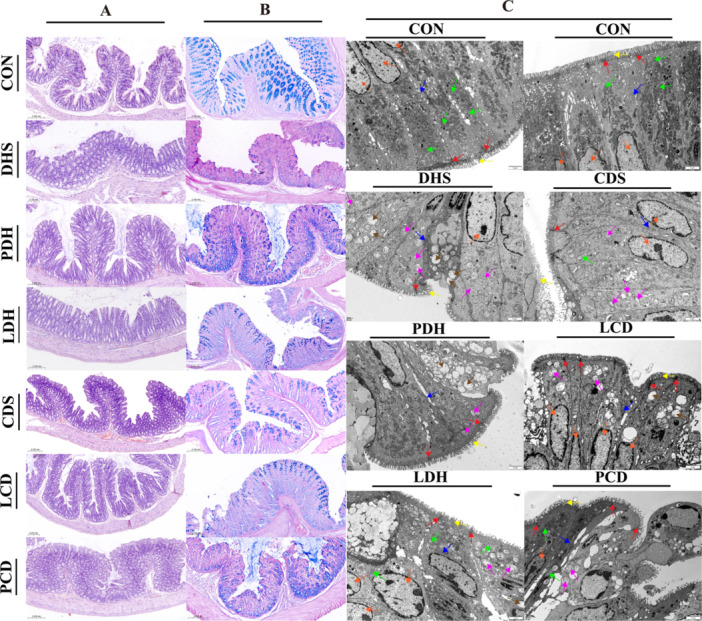
Histopathological evaluation and ultrastructural observation of colonic tissue. (A) Colonic tissue H&E staining sections (100×), (B) colonic tissue AB‐PAS staining sections (100×), and (C) ultrastructural observation of colonic epithelial cells (5000×); Microvilli (yellow arrows), tight junctions (red arrows), mitochondria (green arrows), rough endoplasmic reticulum (blue arrows), cell nucleus (orange arrows), mitochondrial autophagy (brown arrows), swollen mitochondria (pink arrows). AB‐PAS, Alcian Blue‐Periodic Acid‐Schiff; CDS, Cold‐Damp Syndrome; CON, control; DHS, Damp‐Heat Syndrome; H&E, hematoxylin and eosin; LCD, Lizhong decoction Treatment for Cold‐Damp Diarrhea Group; LDH, Lizhong decoction Treatment for Damp‐Heat Diarrhea Group; PCD, Pulsatilla decoction Treatment for Cold‐Damp Diarrhea Group; PDH, Pulsatilla decoction Treatment for Damp‐Heat Diarrhea Group.

The histopathological evaluation results (Figure 5A) indicated that the intestinal epithelial structure of the CON group was intact and well‐defined, characterized by deep crypts and a substantial presence of goblet cells, predominantly located in the basal layer. In contrast to the CON group, colonic tissue from the DHS group exhibited submucosal edema and thickening, along with destruction of the mucosal layer architecture. This damage manifested as injury to both the mucosal and submucosal layers of the colon, degeneration and shedding of epithelial cells, extensive infiltration by inflammatory cells, dissolution and loss of crypts, as well as a marked reduction in goblet cell numbers (Figure 5B).

Electron microscopy revealed necrosis in some mucosal epithelial cells, with intestinal villi appearing dissolved and reduced electron density. Numerous swollen mitochondria were observed within glandular epithelial cell cytoplasm, alongside a significant decrease in tight junction structures between epithelial cells (Figure 5C). In the CDS group, colonic epithelial structure showed more severe changes: a thinner mucosal layer with diminished crypt depth and extensive inflammatory cell infiltration. Electron microscopy examination confirmed disintegrated intestinal villi with decreased electron density; many swollen mitochondria were present in the cytoplasm, along with a notable reduction in tight junction numbers between epithelial cells. After treatment with Pulsatilla decoction and Lizhong decoction, the PDH group showed reduced inflammatory cell infiltration, restored mucosal thickness, deeper crypts, and partial recovery in goblet cell numbers and distribution. Electron microscopy revealed intact colonic epithelial structures; intestinal villi began to recover, tight junctions were relatively complete, and only a few mitochondria in columnar cells exhibited mild swelling. The LDH group displayed histopathological changes similar to the DHS group with no significant improvement. Electron microscopy showed disorganized cytoplasmic structures without notable enhancement. In contrast, the LCD group had reduced inflammation along with some recovery in crypt depth and more regular goblet cell distribution despite their decreased numbers. Electron microscopy indicated normal levels of intestinal villi; electron density increased significantly alongside a rise in tight junctions. The PCD group also exhibited reduced inflammation but did not show significant recovery of crypt integrity or tissue structure. Some improvement was noted in goblet cell numbers; however, they remained irregularly distributed. Minimal differences were observed between the PCD and CDS groups, both had disordered intestinal epithelial structures with no substantial improvements regarding villus integrity or tight junction presence within the intestine.

### 3.3 Detection of Cytokines

**Figure 6:**
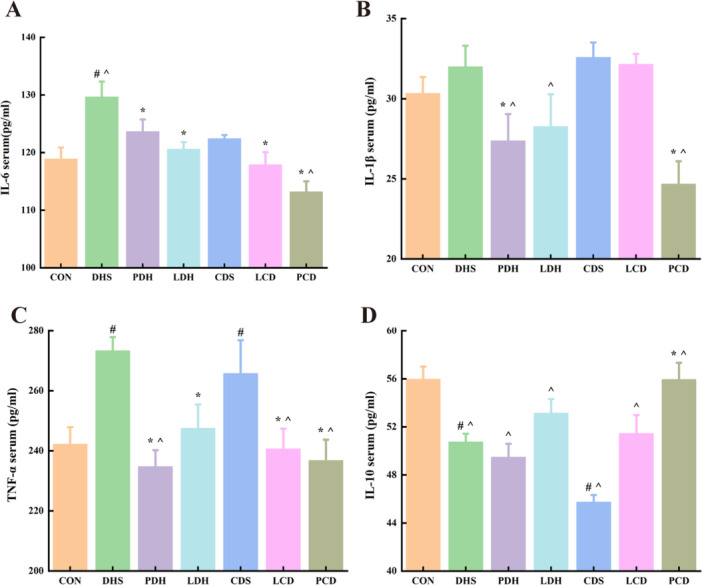
Serum cytokine levels. (A) IL‐6, (B) IL‐1β, (C) TNF‐α, and (D) IL‐10; ^#^ *p* < 0.05 versus control group; *p < 0.05 versus DHS group; ^*p* < 0.05 versus CDS group. CDS, Cold‐Damp Syndrome; CON, control; DAI, Disease Activity Index; DHS, Damp‐Heat Syndrome; IL‐6, interleukin 6; LCD, Lizhong decoction Treatment for Cold‐Damp Diarrhea Group; LDH, Lizhong decoction Treatment for Damp‐Heat Diarrhea Group; PCD, Pulsatilla decoction Treatment for Cold‐Damp Diarrhea Group; PDH, Pulsatilla decoction Treatment for Damp‐Heat Diarrhea Group; TNF‐α, tumor necrosis factor‐α.

The concentrations of IL‐6, IL‐1β, TNF‐α, and IL‐10 in serum are presented in Figure 6A–D. Following the modeling of damp‐heat diarrhea (DHS), the levels of IL‐6, IL‐1β, and TNF‐α in the DHS group were significantly elevated compared with those in the control (CON) group; conversely, the level of IL‐10 was markedly reduced relative to that observed in the CON group (*p* < 0.05). Subsequent intervention with Pulsatilla decoction resulted in a reversal of trends for IL‐6, TNF‐α, and IL‐1β levels when compared with the DHS group. In contrast, after modeling cold‐damp diarrhea (CDS), changes in levels of IL‐6, IL‐1β, TNF‐α as well as that of IL‐10 within the CDS group mirrored those seen in the DHS group. Intervention with Lizhong decoction effectively reversed alterations in levels of IL‐6, TNF‐α, and IL‐1β within the PCD group (*p* < 0.05); however, it did not exert a significant effect on these cytokine levels within the LCD group. These findings indicate that rat models for both damp–heat diarrhea and cold–damp diarrhea have been successfully established.

### 3.4 16S rRNA Sequencing Analysis of Gut Microbiota Diversity and Community Structure in Rat Cecal Contents

**Figure 7:**
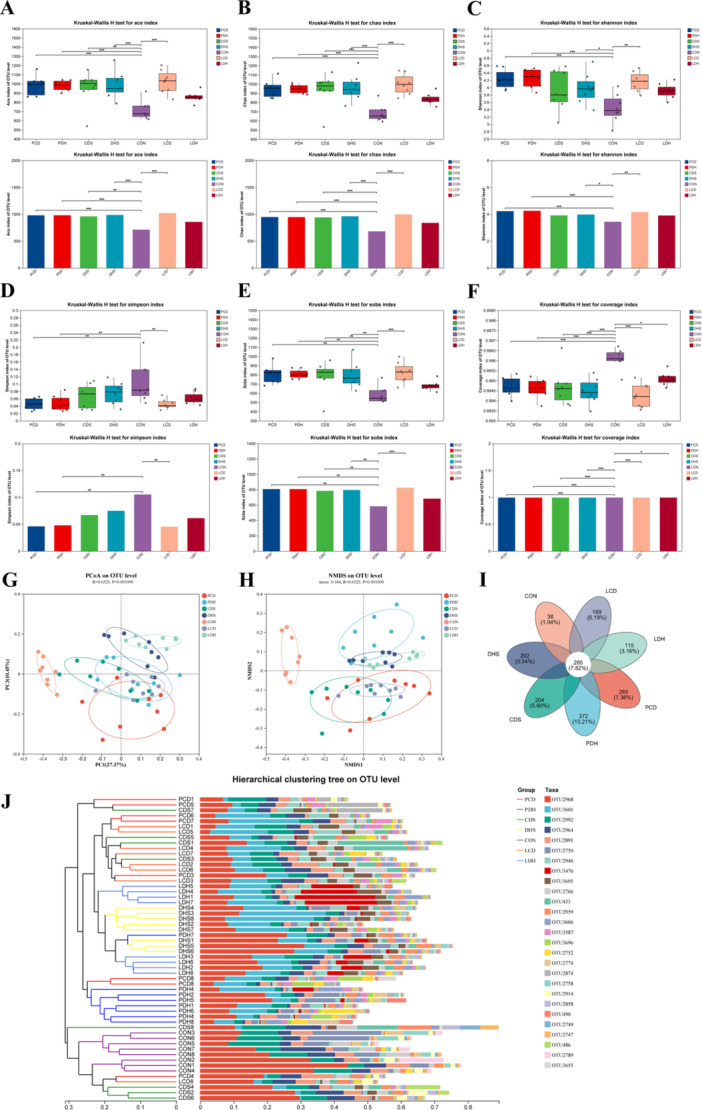
Analysis of gut microbiota α and β diversity and sample hierarchical clustering. (A) Ace index, (B) Chao index, (C) Shannon index, (D) Simpson index, (E) Sobs index, (F) Coverage index, (G, H) PcoA and NMDS analysis, (I) Venn diagram, and (J) Sample hierarchical clustering diagram. *p < 0.01; **p < 0.001; ***p < 0.0001. CDS, Cold‐Damp Syndrome; CON, control; DHS, Damp‐Heat Syndrome; LCD, Lizhong decoction Treatment for Cold‐Damp Diarrhea Group; LDH, Lizhong decoction Treatment for Damp‐Heat Diarrhea Group; NMDS, nonmetric multidimensional scaling; PCD, Pulsatilla decoction Treatment for Cold‐Damp Diarrhea Group; PCoA, principal coordinate analysis; PDH, Pulsatilla decoction Treatment for Damp‐Heat Diarrhea Group.

The 16S rRNA sequencing analysis revealed significant changes in the gut microbiota α‐diversity of both the DHS and CDS rat groups compared with the CON group. After Pulsatilla decoction intervention, various indicators in the PDH and PCD groups were partially restored. Similarly, Lizhong decoction treatment led to some restoration in the LDH group; however, no significant effect was observed on the LCD group (Figure 7A–F). For β‐diversity, PCA, NMDS analysis, and hierarchical clustering showed notable alterations across groups. A clear separation was evident between DHS and CDS groups relative to CON. In contrast, smaller separations were noted among PDH, LDH, LCD, and PCD when compared with controls. This indicates that both Pulsatilla and Lizhong decoctions resulted in less pronounced differences from CON; larger discrepancies were found between DHS/CDS versus CON (Figure 7G,H). Figure 6I illustrates community composition variations: The CON group had 38 OTUs while DHS and CDS contained 202 and 204 OTUs, respectively; PDH and LCD had 372 and 189 OTUs, respectively—LDH comprised 115 OTUs while PCD included 269 OTUs correspondingly.

**Figure 8:**
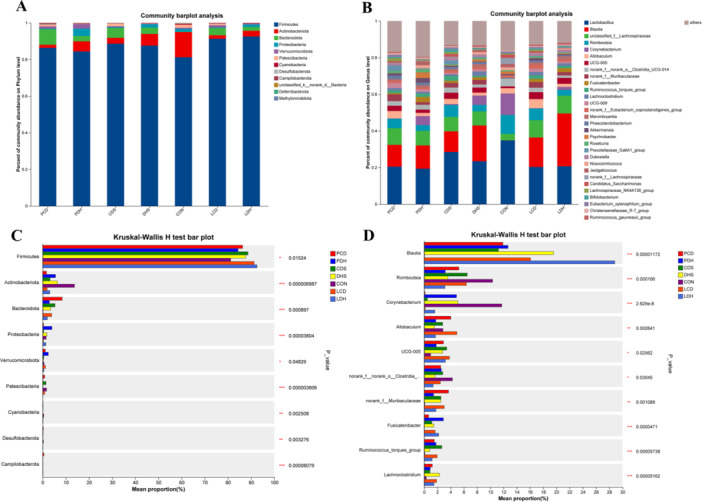
Changes in the structure of gut microbial communities at the phylum and genus levels. (A) Phylum level microbial community structure diagram, (B) Genus level microbial community structure diagram, (C) Significance test of intergroup differences at the phylum level, and (D) Significance test of intergroup differences at the genus level. Data are represented as mean ± SD (standard error of the mean) (*n* = 8). *p < 0.05; **p < 0.01; ***p < 0.001. (C, D) Red is the PCD group, dark blue is the PDH group, green is the CDS group, yellow is the DHS group, purple is the CON group, red is the LCD group, and light blue is the LDH group. CDS, Cold‐Damp Syndrome; CON, control; DHS, Damp‐Heat Syndrome; LCD, Lizhong decoction Treatment for Cold‐Damp Diarrhea Group; LDH, Lizhong decoction Treatment for Damp‐Heat Diarrhea Group; PCD, Pulsatilla decoction Treatment for Cold‐Damp Diarrhea Group; PDH, Pulsatilla decoction Treatment for Damp‐Heat Diarrhea Group.

We analyzed the microbial community composition within each group (Figure 8A,B). At the phylum level, after damp‐heat diarrhea modeling, we noted an increase in Firmicutes and a decrease in Patescibacteria and the Firmicutes/Bacteroidota (F/B) ratio in the DHS group compared with CON. Pulsatilla decoction reversed this trend, while Lizhong decoction showed no improvement. In contrast, following cold‐damp diarrhea modeling, Bacteroidota increased and the F/B ratio decreased in the CDS group. Lizhong decoction successfully reversed these changes; however, Pulsatilla decoction had no significant effect. At the genus level post‐damp‐heat diarrhea modeling, *Lachnoclostridium* increased in relative abundance in DHS versus CON. Again, Pulsatilla decoction countered this trend while Lizhong decoction showed no improvement. After cold‐damp diarrhea modeling, *Ruminococcus*, and *Roseburia* decreased in relative abundances within CDS. Here, Lizhong decoction reversed these changes, whereas Pulsatilla decoction did not significantly impact them. Figure 8C shows significance tests for intergroup differences at both phylum and genus levels; notable bacterial phyla and genera are marked as significantly different (*p* < 0.01) or extremely significantly different (*p* < 0.001). Thus, both Pulsatilla decoction and Lizhong decoction have effectively improved gut microbiota structure and diversity in rats with damp‐heat or cold‐damp diarrhea.

### 3.5 Content of SCFAs in the Intestinal Contents of Diarrhea Rats and Expression of SCFA Receptors

**Figure 9:**
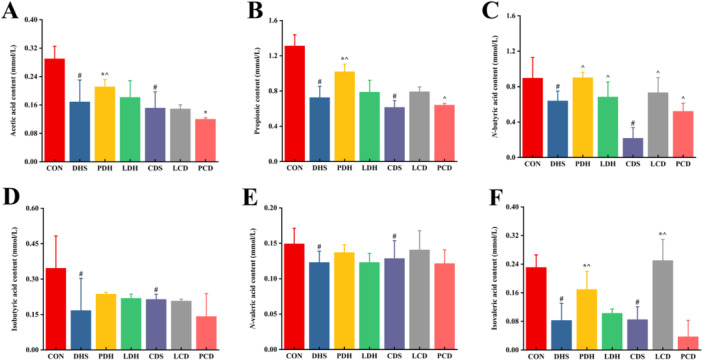
Content of SCFAs in cecal contents. (A) Acetic acid, (B) propionic acid, (C) butyric acid, (D) isobutyric acid, (E) valeric acid, and (F) isovaleric acid; Data shown as mean ± SD (*n* = 6), ^#^ *p* < 0.05 indicates comparison with the control group, *p < 0.05 indicates comparison with the DHS group, ^*p* < 0.05 indicates comparison with the CDS group. CDS, Cold‐Damp Syndrome; CON, control; DHS, Damp‐Heat Syndrome; LCD, Lizhong decoction Treatment for Cold‐Damp Diarrhea Group; LDH, Lizhong decoction Treatment for Damp‐Heat Diarrhea Group; PCD, Pulsatilla decoction Treatment for Cold‐Damp Diarrhea Group; PDH, Pulsatilla decoction Treatment for Damp‐Heat Diarrhea Group; SCFAs, short‐chain fatty acids.

**Figure 10:**
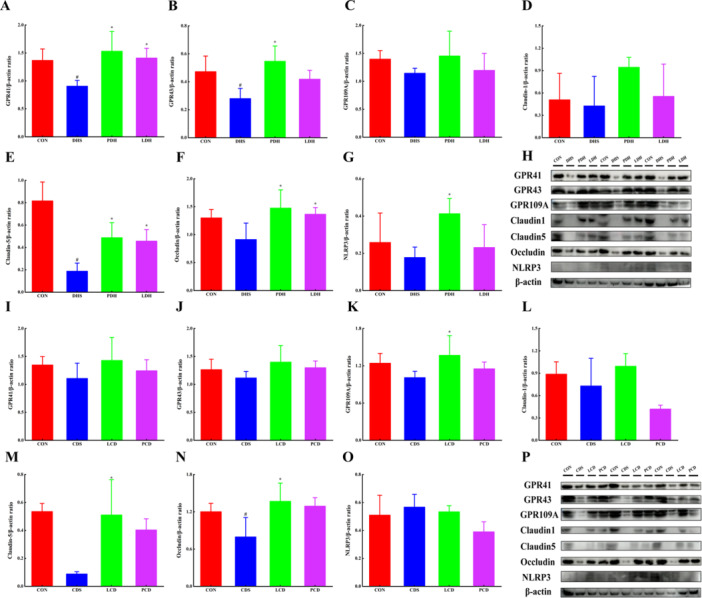
Expression of G protein‐coupled receptors GPR 41, GPR 43, GPR 109A, and intestinal tight junction proteins in rat colonic tissue. (A) GPR 41, (B) GPR 43, (C) GPR 109A, (D) Claudin1, (E) Claudin5, (F) Occludin, (G) NLRP3, (H) WB results of damp‐heat diarrhea, (I) GPR 41, (J) GPR 43, (K) GPR 109A, (L) Claudin1, (M) Claudin5, (N) Occludin, (O) NLRP3, and (P) WB results of cold‐damp diarrhea. Changes in the relative expression of Claudin1, Claudin5, Occludin, and inflammasome NLRP3. Data shown as mean ± SD (*n* = 3), ^#^ indicates comparison with the control group *p* < 0.05, * indicates comparison with the DHS group or CDS group *p* < 0.05. CDS, Cold‐Damp Syndrome; CON, control; DHS, Damp‐Heat Syndrome; LCD, Lizhong decoction Treatment for Cold‐Damp Diarrhea Group; LDH, Lizhong decoction Treatment for Damp‐Heat Diarrhea Group; PCD, Pulsatilla decoction Treatment for Cold‐Damp Diarrhea Group; PDH, Pulsatilla decoction Treatment for Damp‐Heat Diarrhea Group.

The content of SCFAs in the intestinal contents of each group of rats is presented in Figure 9A–F. Compared with the CON group, SCFAs levels were significantly reduced across all model groups. Following the modeling of damp‐heat diarrhea, Pulsatilla decoction effectively reversed the decline in n‐butyric acid and isobutyric acid levels; however, Lizhong decoction did not demonstrate any improvement in this model. In contrast, after modeling cold‐damp diarrhea, Lizhong decoction successfully restored n‐butyric acid and isobutyric acid levels, while Pulsatilla decoction exhibited limited effects on the cold‐damp diarrhea model. In Figure 10, relative expression levels of GPR 41, GPR 43, GPR 109 A, Occludin, Claudin1, Claudin5, and NLRP3 proteins were significantly decreased in both the DHS group following damp‐heat diarrhea modeling and the CDS group following cold‐damp diarrhea modeling when compared with the CON group. After intervention with Pulsatilla decoction and Lizhong decoction, these protein expression levels were partially restored to varying degrees.

### 3.6 Correlation Analysis Between SCFAs and Gut Microbiota

**Figure 11:**
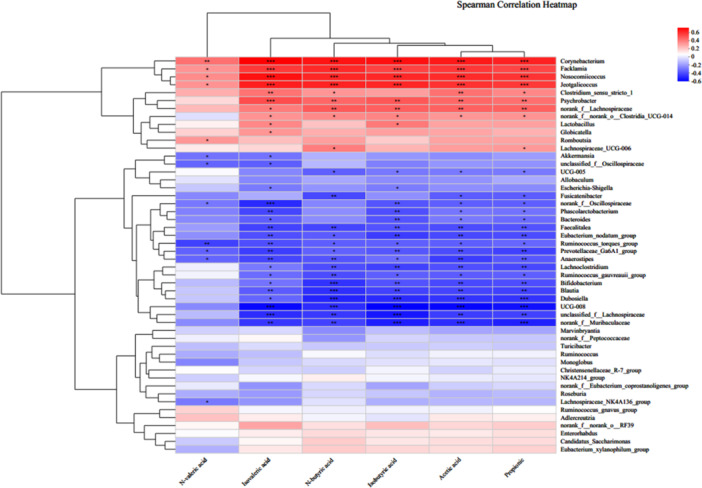
Correlation analysis between significantly different bacteria and significantly different metabolites.

The results of the correlation analysis between significantly different bacteria and SCFAs (Figure 11) revealed that acetic acid, propionic acid, isobutyric acid, n‐butyric acid, and isovaleric acid exhibited extremely significant positive correlations with Bacillus, Faecalibacterium, nosocomiicoccus, and Psychrobacter. Conversely, these fatty acids showed significant negative correlations with *Koalaibacterium*, *Bacteroides*, *Prevotella*, *Anaerostipes*, *Bifidobacterium*, *Dorea*, *Blautia*, *Lachnoclostridium*, and *Muribaculaceae*. Notably, Clostridium butyricum demonstrated a positive correlation with acetic acid, propionic acid, and isovaleric acid; *Lactobacillus* was significantly positively correlated with both isovaleric acid and isobutyric acid; while *Roseburia* exhibited a significant positive correlation with acetic acid. These findings suggest that Pulsatilla decoction and Lizhong decoction may exert therapeutic effects in cases of damp‐heat and cold‐damp diarrhea by restoring the structure of gut microbiota as well as the levels of SCFAs.

The right side of the heatmap is the microbial classification, and the bottom is the SCFAs. The left and top sides are the clustering dendrograms based on the correlation coefficient. The color bar represents the magnitude of the correlation coefficient, the closer the absolute value is to 1, the higher the correlation between metabolites, red indicates positive correlation, and blue indicates negative correlation. The deeper the color, the stronger the correlation, and the asterisk indicates a significant correlation between metabolites and microbes: *p < 0.05; **p < 0.01; ***p < 0.001.

## 4 Discussion

In this study, the results showed that rats in the Damp‐Heat Syndrome (DHS) and Cold‐Damp Syndrome (CDS) groups experienced weight loss, severe bloody stools, reduced food and water intake, increased rates of loose stools and diarrhea indices, significantly increased DAI scores, elevated levels of inflammatory factors, severe colonic tissue pathology, a marked decrease in the number of goblet cells and tight junction proteins are markedly reduced (Table 3). After treatment, these indicators improved. However, the surprising finding in this study is that there are significant differences in the changes in gut microbiota and SCFAs content between the two different syndrome types, which may be used for clinical dialectic. The results of this study indicate that mucosal color have the potential for clinical diagnosis of damp‐heat diarrhea and cold‐damp diarrhea. Moreover, *Lachnoclostridium* and *Marvinbryantia* are potential biomarkers for distinguishing between damp‐heat diarrhea and cold‐damp diarrhea (Table 4).

**Table 3:** Comparison table of groups.

Group name		CON vs. DHS	CON vs. CDS	CDS vs. DHS
Mucosal color		Red	Purple	RED
Transmission electron microscopy		Mitochondrial swelling	Tight junction strengthening	Mitochondrial swelling
Histopathological	Intestinal mucosal epithelium	Hyperemia	Inflammatory cell infiltration	Hyperemia
	Goblet cell	Down	Down	Down
Cytokines	IL‐6	Up	Up	Up
	IL‐1β	Up	Up	Down
	TNF‐α	Up	Up	Up
	IL‐10	Down	Down	Up
Tight junction	Occludin	Down	Down	Up
	Claudin1	Down	Down	Up
	Claudin5	Down	Down	Up
SCFAs	Butyric acid	Down	Down	Down
	Isovaleric acid	Down	Down	Down
SCFAs receptor	GPR41	Down	Down	Down
	GPR43	Down	Down	Down
	GPR109A	Down	Down	Up
Gut microbiota	F/B	Down	Down	Up
	Firmicutes	Up	Up	Down
	Bacteroidota	Up	Up	Down
	Actinobacteriota	Down	Down	Up
	Proteobacteria	Up	Down	Up
	*Lachnoclostridium*	Up	Up	Up
	*Marvinbryantia*	Up	Up	Up
	*Akkermansia*	Down	Down	Up

Abbreviations: CDS, Cold‐Damp Syndrome; CON, control; DHS, Damp‐Heat Syndrome; F/B, Firmicutes/Bacteroidota; IL‐6, interleukin 6; SCFAs, short‐chain fatty acids; TNF‐α, tumor necrosis factor‐α.

**Table 4:** Weight versus body temperature comparison chart.

Groups	Temperature (°C)		Weight(g)	
	Before treating	After treating	Before treating	After treating
CON	37.6±0.1	36.3±0.2	226.5±5.2	244.2±4.8*
DHS	37.3 ± 0.1	39.0 ± 0.2*	221.6 ± 4.8*	180.2 ± 3.7*
PDH	37.8 ± 0.2	38.8 ± 0.2*	198.6 ± 4.0	178.9 ± 3.6*
LDH	37.1 ± 0.2	38.8 ± 0.2*	225.5 ± 6.0	173.4 ± 5.6*
CDS	36.9 ± 0.2*	35.9 ± 0.3	227.4 ± 5.3	220.4 ± 6.0*
LCD	37.2 ± 0.1*	36.2 ± 0.2	216.8 ± 7.5	218.9 ± 4.5*
PCD	37.3 ± 0.1	36.2 ± 0.2	218.9 ± 4.7	212.1 ± 4.7*

We concentrated on identifying the signals that differentiate between the damp‐heat diarrhea and cold‐damp diarrhea groups, as clinical symptoms may overlap between these two conditions; however, their treatments differ significantly. In cases of worsening damp‐heat diarrhea, clinicians may increase electrolyte supplementation to combat dehydration, whereas different therapeutic approaches are typically employed for cold‐damp diarrhea. We utilized Linear Discriminant Analysis (LDA) to assess potential differentiating factors in intestinal microbiota classification and conducted Spearman correlation analysis on SCFA content data (Figure 10). The most effective distinguishing factor between damp‐heat diarrhea and cold‐damp diarrhea is the classification of intestinal microbiota. Evidently, the impacts of damp‐heat diarrhea and cold‐damp diarrhea on the microbiome exhibit considerable heterogeneity. Furthermore, both groups experience extensive exposure to high‐humidity environments, which may similarly influence community composition within each group. Concurrently, clinical symptom observations reveal marked differences between damp‐heat and cold‐damp diarrhea. The mucosa of rats with damp‐heat diarrhea appears red and rosy; additionally, mitochondria in their intestinal epithelial cells display signs of swelling or autophagy. In contrast, rats suffering from cold‐damp diarrhea exhibit a purple‐red mucosa along with a significant reduction in tight junctions among intestinal epithelial cells.

Alterations in the composition of the intestinal microbiota are closely associated with the onset of various diseases, including colitis, obesity, and diabetes (Francis et al. 2023). Among these conditions, colonic inflammation is regarded as a consequence of an inappropriate response from the mucosal immune system triggered by the intestinal microbiota(Maldonado‐Arriaga et al. 2021). This response is characterized by an increase in potentially harmful bacteria and a decrease in beneficial bacterial populations (Haq et al. 2019). A previous study has demonstrated that modifications in the structure of the intestinal microbiota can exacerbate mild mucosal inflammation (Burrello et al. 2019). The analysis of taxonomic data regarding the intestinal microbiota revealed significant differences between the damp‐heat diarrhea group and the cold‐damp diarrhea group, particularly in three bacterial taxa: *Lachnoclostridium*, *Marvinbryantia*, and *Akkermansia*. Notably, this finding has not been documented in prior research. In the damp‐heat diarrhea group, levels of *Lachnoclostridium* and *Marvinbryantia* were relatively low; however, these levels gradually increased following successful treatment. In contrast, within the cold‐damp diarrhea group, *Lachnoclostridium* and *Marvinbryantia* were initially higher but exhibited a gradual decrease with effective intervention. *Akkermansia* was found to be at relatively low levels in both the damp‐heat and cold‐damp diarrhea groups but showed an increase with successful treatment. Additionally, previous studies have indicated that Firmicutes and Bacteroidetes are essential constituents of the human microbiome (An et al. 2023; Magne et al. 2020; Yin et al. 2025). The Firmicutes phylum plays a crucial role in limiting the proliferation of pathogenic bacteria (Xu et al. 2018). Its subphylum, *Lachnoclostridium*, is known to suppress inflammation and produce butyric acid, which aids in repairing the intestinal barrier and promoting overall intestinal health (Huang et al. 2022; Song et al. 2023). Existing research indicates that members of the Clostridium genus, including *Marvinbryantia*, can utilize cellulose and methylcellulose while promoting the production of succinic acid.

In previous studies, a negative correlation has been observed between the concentrations of MDA, IL‐6, TNF‐α, IL‐1β, and MPO with the relative abundance of *Marvinbryantia*. Conversely, this relative abundance displays a positive correlation with SCFAs, including acetic acid, propionic acid, isobutyric acid, butyric acid and isovaleric acid, as well as colon length and antioxidant enzyme activity (GSH and SOD) (Guo et al. 2023, 2022). *Marvinbryantia* significantly inhibits inflammatory cytokine secretion by producing beneficial substances, like, SCFAs. Appropriate SCFAs concentrations have been shown to diminish IL‐1β production by blocking the NLRP3 pathway and modulating Treg cell activity through GPR43 activation (Hu et al. 2016; Zhang et al. 2021). Furthermore, beneficial bacteria such as *Marvinbryantia* can impede harmful bacterial growth by competing for nutrients and space within the intestine; this competition alleviates dysbiosis within colitis rats models while maintaining intestinal barrier integrity. Overall, there exists a positive correlation between *Marvinbryantia* abundance and SCFAs concentration in the cecum while demonstrating an inverse relationship with IL‐1β levels in the colon (Gao et al. 2022; Guo et al. 2023). *Akkermansia muciniphila* is recognized as a beneficial intestinal bacterium. The supplementation of *A. muciniphila* has been shown to alleviate symptoms of enteritis, regulate immune responses, reduce levels of inflammation within the intestine, and prevent intestinal cancer induced by enteritis. Prior studies have demonstrated that in the DSS mouse colitis model, *A. muciniphila* exhibits notable anti‐inflammatory effects (Zhai et al. 2019; Ke et al. 2021). Furthermore, this study reported a reduction in cytokine and chemokine levels associated with its application. Additionally, research conducted by Ottman et al. identified that *A. muciniphila* plays a crucial role in enhancing host immune homeostasis and improving intestinal barrier function within the intestinal mucosa (Chang et al. 2022; Pei et al. 2023). Recent investigations further corroborate these findings by highlighting improvements in intestinal health through the enhancement of gut integrity and maintenance of immune homeostasis (Ma et al. 2024; T. Li, Yu, et al. 2024). Neutrophil infiltration serves as a significant marker indicating inflammation beyond cytokine release. Stimulating agents lead to an increase in cytokines and chemokines within the colonic mucosa; subsequently, neutrophils migrate to sites of damage via vascular wall infiltration. Treatment with *A. muciniphila* has been shown to significantly reduce neutrophil infiltration (Lowe et al. 2017). The aforementioned research outcomes align consistently with those presented in this study. The levels of IL‐6, TNF‐α, and IL‐1β were significantly elevated in both the damp‐heat diarrhea group and the cold‐damp diarrhea group. Following treatment, these levels showed a significant decrease in both groups. However, no statistically significant difference was observed between the two groups.

The results obtained from this investigation hold biological significance for diagnosing and treating diarrheal diseases. This experiment additionally screened *Lachnoclostridium* and *Marvinbryantia* as potential biomarkers to differentiate between damp‐heat diarrhea and cold‐damp diarrhea. They possess a compelling biological basis as candidates for such applications. We propose that evaluating the abundance of *Lachnoclostridium* and *Marvinbryantia* may be instrumental in diagnosing damp‐heat diarrhea versus cold‐damp diarrhea when used as disease markers.

Although this study successfully validated the therapeutic effects of Pulsatilla and Lizhong decoctions on the damp‐heat diarrhea and cold‐damp diarrhea model at the whole‐animal level, as evidenced by significant improvements in key phenotypes such as the diarrhea index and intestinal inflammation score, their precise molecular and cellular mechanisms remain to be fully elucidated. On the basis of our findings and existing literature, we herein discuss their potential levels of action. First, Pulsatilla decoction exhibited notable anti‐inflammatory activity, suggesting its action may reside at the cellular and molecular levels (Niu et al. 2023). Furthermore, the possibility that it acts by modulating the gut microbiota structure cannot be overlooked. On the other hand, Lizhong decoction administration promotes Th17/Treg cell balance in a gut microbiota‐SCFAs‐dependent manner, which in turn ameliorates Inflammation (Huang et al. 2025).

Lizhong decoction remarkably improved intestinal pathological injury in UC mice, and its potential mechanism was the suppression of ferroptosis in enterocytes by the Nrf2/SLC7A11/GPX4 pathway (W. Li, Wang, et al. 2024). Lizhong decoction can improve infection by regulating the intestinal physical barrier and immune response (Miao et al. 2023). In summary, we hypothesize that Pulsatilla and Lizhong exert their therapeutic effects via multilevel and multitarget pathways, primarily characterized by “anti‐inflammatory/microbiota modulation” and “barrier repair,” respectively. Elucidating their precise molecular targets constitutes a key focus for our future research, which plans to utilize advanced technologies such as metabolomics and proteomics for in‐depth mechanistic exploration.

This study presents several limitations. First, the research subjects were rat models, which may introduce heterogeneity in various clinical features and complex individual differences among different rats. Second, the assessment of mucosal color, based on TCM inspection theory, remained subjective in this study. The lack of objective quantification precluded establishing a precise correlation between specific color ranges and the two diarrhea syndromes. Future research should focus on developing objective measurement techniques and conducting large‐scale clinical trials to validate these observations. The current research will aid in addressing these limitations in future investigations.

## 5 Conclusion

There are differences in gut microbiota dysbiosis among different TCM syndrome types of diarrhea. Mucosal color and histopathological observations have the potential for clinical diagnosis of damp‐heat diarrhea and cold‐damp diarrhea. Moreover, *Lachnoclostridium* and *Marvinbryantia* are potential biomarkers for distinguishing between damp‐heat diarrhea and cold‐damp diarrhea. However, the *Lachnoclostridium and Marvinbryantia* Validation and clinical application require further investigation.

## Author Contributions

**Hao Zhang:** investigation, methodology, writing – original draft. **Xia Song:** writing – review and editing. **Wenwen Mi:** methodology, data curation, validation. **Peng Ji:** formal analysis, resources. **Yanming Wei:** conceptualization. **Yongli Hua:** funding acquisition, project administration, writing – review and editing.

## Ethics Statement

This study was approved by the ethics committee of Gansu Agricultural University (approval GSAU‐Eth‐VMC‐2023‐040). We certify that the study was performed in accordance with the 1964 declaration of HELSINKI and later amendments.

## Consent

All participants involved in this study provided their informed consent for the publication of anonymized data.

## Conflicts of Interest

None declared.

## Data Availability

All the data required for this article will be provided upon request.
